# Changes in cytochrome P450s-mediated drug clearance in patients with hepatocellular carcinoma *in vitro* and *in vivo*: a bottom-up approach

**DOI:** 10.18632/oncotarget.8704

**Published:** 2016-04-12

**Authors:** Jie Gao, Jun Zhou, Xiao-Pei He, Yun-Fei Zhang, Na Gao, Xin Tian, Yan Fang, Qiang Wen, Lin-Jing Jia, Han Jin, Hai-Ling Qiao

**Affiliations:** ^1^ Institute of Clinical Pharmacology, Zhengzhou University, Zhengzhou, China

**Keywords:** hepatocellular carcinoma, bottom up IVIVE approach, hepatic clearance, cytochrome P450s, microsomal protein per gram of liver

## Abstract

Hepatocellular carcinoma (HCC) accompanied by severe liver dysfunction is a serious disease, which results in altered hepatic clearance. Generally, maintenance doses depend upon drug clearance, so individual dosage regimens should be customized for HCC patients based on the condition of patients. Based on clearance of CYP isoform-specific substrates at the microsomal level (CL_M_), microsomal protein per gram of liver (MPPGL), liver weight, hepatic blood flow, hepatic clearance values (CL_H_) for 10 CYPs in HCC patients (n=102) were extrapolated using a predictive bottom-up pharmacokinetic model. Compared with controls, the CL_M_ values for CYP2C9, 2D6, 2E1 were significantly increased in HCC patients. Additionally, CYP1A2, 2C8, 2C19 CL_M_ values decreased while the values for CYP2A6, 2B6, 3A4/5 were unchanged. The MPPGL values in HCC tissues were significantly reduced. CL_H_ values of HCC patients for CYP1A2, 2A6, 2B6, 2C8, 2C19, and 3A4/5 were significantly reduced, while this for CYP2E1 were markedly increased and those for CYP2C9 and 2D6 did not change. Moreover, disease (fibrosis and cirrhosis) and polymorphisms of the CYP genes have influenced the CL_H_ for some CYPs. Prediction of the effects of HCC on drug clearance may be helpful for the design of clinical studies and the clinical management of drugs in HCC patients.

## INTRODUCTION

Significant research efforts towards the development of personalized medicine have been conducted especially for patients with liver diseases that usually are accompanied by the loss of functional hepatocytes. Because of the complex pharmacokinetics changes that occur in liver diseases, both the US FDA and European Medicines Agency have released general guidelines that recommend conducting pharmacokinetic studies when drugs are likely to be used in patients with impaired hepatic function.

Hepatocellular carcinoma (HCC) is the most common type of liver cancer and represents a leading cause of cancer death worldwide [1]. HCC often results in biochemical and physiological changes, such as altered hepatic function, hepatic blood flow (Q_H_), functional liver size, and plasma protein binding. These changes can result in changed clearance compared with what is observed in subjects with normal hepatic function. Generally, maintenance doses depend on drug clearance, so individual dosage regimens should be customized for HCC patients based on the condition of each patient.

Cytochrome P450 (CYP) represents a large group of enzymes that localize to the endoplasmic reticulum and play critical roles in the metabolism of endogenous and exogenous molecules, including most carcinogens, procarcinogens and drugs [2]. Previous studies of CYP have found that the clearance for CYP3A4 in tumor tissues from HCC patients was significantly reduced compared with adjacent non-cancerous tissues [3]. Additionally, *in vitro* studies have indicated that clearance values for CYPs were selectively altered in the presence of cirrhosis [4, 5]. Moreover, previously published pharmacokinetic studies have demonstrated that clearance for CYP3A4/5 was markedly decreased in patients with either cirrhosis [6] or severe alcoholic cirrhosis [7], while the clearance for CYP2C19 was also significantly reduced in patients with liver cirrhosis [8]. Therefore, assessments of changes in clearance values for CYPs may be useful not only for designing personalized HCC treatments, but also for identifying dosage regimens for drugs that are used to treat HCC patients who suffer from other diseases. However, studies that base dosage adjustments on changes in clearance values for CYPs in HCC patients have not been previously reported.

To customize individual dosage regimens for HCC patients with scarce or imprecise available data, *in vitro* studies using human liver microsomes (HLMs) from the patients combined with predictive bottom-up pharmacokinetic models should be employed. A study by Johnson *et al.* suggested that Q_H_, functional liver size, and plasma protein binding were altered in correlation with the severity of liver cirrhosis [9]. Therefore, changes in these parameters for HCC patients might result in different changes in drugs clearance, both *in vitro* and *in vivo*, compared with controls. Indeed, other than Q_H_, functional liver size, and plasma protein binding, the change in microsomal protein per gram of liver (MPPGL) has been found to be a more important parameter. Unfortunately, data regarding MPPGL values in patients with liver cirrhosis or HCC have not been reported, which represents a serious obstacle to determining the CL_H_ in HCC patients.

Accordingly, an *in vitro* study of 102 HCC patient samples was performed that focused on the clearance changes for 10 CYPs–CYP1A2, 2A6, 2B6, 2C8, 2C9, 2C19, 2D6, 2E1, and 3A4/5. Relevant physiological and biochemical changes related to liver disease were incorporated into predictive bottom-up pharmacokinetic models to analyze changes in clearance at different levels. The effects of an accompanying disease (i.e., cirrhosis and/or fibrosis), genetic polymorphisms, and demography on hepatic clearance were evaluated. We hope the findings of this study could be applied to guide appropriate trial design for population pharmacokinetic studies or the clinical management of drugs in HCC patients in whom no clinical data exist.

## RESULTS

### Clearance at the microsomal level

**Figure 1:**
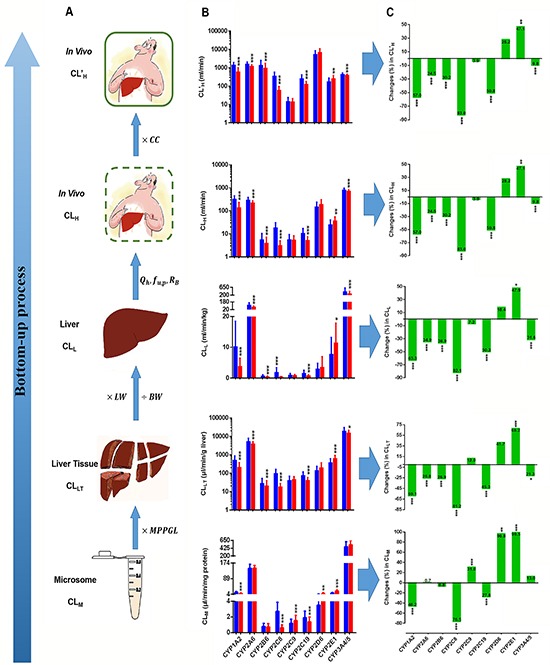
The bottom-up process (A), Clearance for CYPs at different levels in hepatocellular carcinoma (HCC) patients and control subjects (B) and changes in clearance rates at different levels in HCC patients (C) CL_M_: clearance at the microsomal level; MPPGL: microsomal protein per gram of liver; CL_LT_: clearance at the liver tissue level; LW: liver weight; BW: body weight; CL_L_: clearance at the liver level; Q_H_: hepatic blood flow; f_u, p_: fraction unbound in plasma; R_B_, ratio of the drug concentration in blood to plasma; CL_H_: clearance *in vivo*; CC: correction coefficient; CL’_H_: corrected clearance *in vivo*. The clearance for CYPs are expressed as medians with the inter-quartile range. A blue bar represents clearance in controls; a red bar represents clearance in the HCC group. “*”, “**”, and “***” indicate significant differences from controls (*P*<0.05, *P*<0.01, and *P*<0.001, respectively) by the Mann–Whitney U test.

Clearance determined based on “per mg of microsomal protein” was considered as CL_M_, which was calculated based on the ratio of V_max_ to K_m_. The CL_M_ values for 10 CYPs (CYP1A2, 2A6, 2B6, 2C8, 2C9, 2C19, 2D6, 2E1, and 3A4/5) were measured in HLMs from both HCC groups and controls; the results are shown in Figure 1.

Compared with controls, the CL_M_ values for CYP2C9, 2D6, and 2E1 increased in HCC patients (*P*=7.96E-5, 2.91E-3, and 1.56E-21, respectively), while those values for CYP1A2, 2C8, and 2C19 were lower (*P*=9.61E-9, 1.19E-24, and 3.61E-4, respectively). The CL_M_ values for 2A6, 2B6, and CYP3A4/5 were unchanged. Notably, the most marked increase was the CL_M_ value for CYP2E1, which increased by 99.5%, while the most prominent reduction was the CL_M_ for CYP2C8, which declined by 77%.

There was great intra-individual variation in the CL_M_ values for CYPs. Notably, the ratios of maximum to minimum were 147.7, 1000.0, 89.0, 100.0, 56.3, 17.7, 111.6, 13.1, and 14.9 for the CL_M_ values for CYP1A2, 2A6, 2B6, 2C8, 2C9, 2C19, 2D6, 2E1, and 3A4/5.

### Clearance at the liver tissue level

The MPPGL values [28.85 (7.60–93.60) mg/g] were initially determined in 102 HCC patients. The MPPGL values in HCC patients were not normally distributed and were significantly lower (*P*=1.48E-5) than the values in control patients[39.60 (9.90–127.90) mg/g] [10].

According to the contents of MPPGL, individual clearance of liver tissue (CL_LT_) for CYPs was calculated by multiplying each individual MPPGL by the corresponding individual CL_M_ for CYPs, which represents CYP-mediated clearance in liver tissue. As shown in Figure 1, the CL_LT_ values for CYP1A2, 2A6, 2B6, 2C8, 2C19, and 3A4/5 in HCC patients were dramatically lower than in controls (*P*=2.00E-11, 7.88E-5, 1.37E-2, 8.86E-26, 1.01E-7, and 1.37E-2, respectively). Notably, the CL_LT_ for CYP2E1 was significantly greater (*P*=3.12E-6). However, the CL_LT_ for CYP2C9 and 2D6 showed no significant difference. Overall, the CL_LT_ for most CYPs were significantly lower in HCC patients.

### Clearance at the liver level

The mean body weight (BW) of the 105 control cases was 63.96 (30.00–92.00) kg, and the mean liver weight (LW) calculated based on the BW of these cases was 1337.24 (912.31–1688.09) g. The mean BW and LW of the102 HCC patients were 66.00 (40.00–101.00) kg and 1103.84 (840.32–1458.57) g, respectively. The LW of the HCC patients was significantly lower than that of the controls (*P*=1.34E-11), while the BW of two groups showed no significant difference.

According to the BW and LW values determined above, individual clearance in the liver (CL_L_) values were obtained by multiplying each individual LW/BW by the individual CL_LT_, which represents CYP-mediated clearance in the liver. As shown in Figure 1, the CL_L_ values for CYP1A2, 2A6, 2B6, 2C8, 2C19, and 3A4/5 in HCC patients were dramatically lower than in controls (*P*=1.04E-13, 1.92E-7, 8.99E-4, 3.77E-27, 6.71E-10, and 3.10E-4, respectively), while the CL_L_ value for CYP2E1 was higher (*P*=1.27E-3) and the CL_L_ value for CYP2C9 and 2D6 showed no significant difference. At the liver level, most CYP CL_L_ values were also markedly lower in HCC patients.

### *In vivo* clearance

The mean C_O_ value for control cases, which was determined based on age and gender, was 5.14 (4.92–6.65) L/min. The mean Q_H_ value calculated based on the C_O_ was 1259.30 (1205.40–1629.25) ml/min. The mean C_O_ and Q_H_ values in HCC patients of 5.83 (4.92–6.65) L/min and 1428.35 (1205.40–1629.25) ml/min were significantly higher than those in controls (*P*=1.26E-10 and 1.29E-10, respectively).

Using the conventional *in vitro*–*in vivo* extrapolation (IVIVE) method, clearance at the *in vivo* level or hepatic clearance (CL_H_) for CYPs in controlled cases and HCC patients were predicted; results are shown in Figure 1. Compared with controls, only the CL_H_ for CYP2E1 in HCC patients was significantly increased (*P*=1.28E-4), while the CL_H_ values for CYP1A2, 2A6, 2B6, 2C8, 2C19, and 3A4/5 were significantly reduced (*P*=6.14E-13, 3.86E-6, 1.46E-3, 9.98E-27, 1.87E-9, and 1.30E-2, respectively) and the CL_H_ for CYP2C9 and 2D6 did not significantly change.

**Figure 2:**
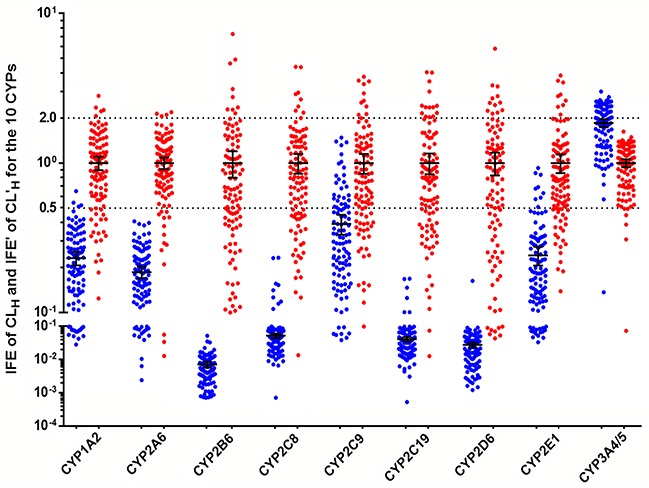
Individual fold-error (IFE) of CLH and corrected individual fold-error (IFE’) of CL'H for the 10 CYPs in control subjects (n=105) $\text{IFE}=10^{\frac{1}{N}\sum \text{ log}(\text{predicted individual value}/\text{observed overall mean})}$; ${\text{IFE}}^{\prime }=10^{\frac{1}{N}\sum \text{ log}(\text{corrected }\mathrm{predictive individual value}/\text{observed overall mean})}$. The blue ball represents the IFE, the red ball represents the IFE’. The black horizontal lines represent the mean with 95% confidence interval.

To evaluate the predictive performance, the accuracy of the predicted CL_H_ values for CYPs in control cases were compared with the observed clearance *in vivo*; results are shown in Figure 2. The AFEs were 0.231 for CYP1A2, 0.186 for CYP2A6, 0.007 for CYP2B6, 0.053 for CYP2C8, 0.391 for CYP2C9, 0.042 for CYP2C19, 0.028 for CYP2D6, 0.241 for CYP2E1, and 1.854 for CYP3A4/5, which demonstrated that only the CL_H_ for CYP3A4/5 was correct. To test the accuracy of each individual measurement, the IFE was calculated. Our findings suggested that the CL_H_ value for CYP3A4/5 matched most closely with its clearance *in vivo*, for which 58 (55.2%) of the cases were within a 2-fold error range. After CYP3A4/5, the CL_H_ value for CYP2C9 matched best with its clearance *in vivo*, for which 28 (26.7%) of the cases were within a 2-fold error range.

**Figure 3:**
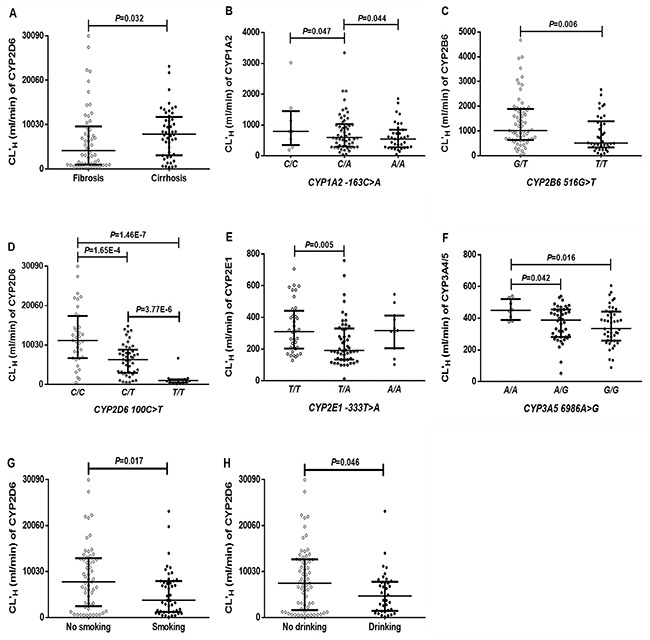
The effects of disease (A), genetic polymorphisms (B–F), smoking (G), and drinking (H), on the corrected predictive hepatic clearance (CL’_H_) for CYPs in HCC patients (n=102) Black horizontal lines represent medians with the inter-quartile range. Disease (fibrosis, n=54; cirrhosis, n=48); *CYP1A2 -163C>A* (*C/C*, n=8; *C/A*, n=54; *A/A*, n=35); *CYP2B6 516G>T* (*G/T*, n=63; *T/T*, n=38); *CYP2D6 100 C>T* (*C/C*, n=35; *C/T*, n=45; *T/T*, n=16); *CYP2E1 -333 T>A* (*T/T*, n=36; *T/A*, n=50; *A/A*, n=11); *CYP3A5 6986 A>G* (*A/A*, n=8; *A/G*, n=42; *G/G*, n=45); smoking (no smoking, n=57; smoking, n=45); alcohol (no drinking, n=65; drinking, n=37).

No data on the *in vivo* clearance for CYPs in HCC patients was identified in a literature search, and only data regarding the clearance for CYP2C19 (67±40 ml/min) and CYP3A4/5 (319.49±154.42 ml/min) in liver cirrhosis were queried. Therefore, the CL_H_ values for CYP2C19 and 3A4/5 predicted using the conventional IVIVE method for HCC patients were compared with the observed clearance in cirrhotic patients; our findings revealed that the AFE values for CYP2C19 and 3A4/5 were 0.087 and 2.111, respectively.

To obtain more accurate measurements of the CL_H_ value for CYPs, the CCs of different CYPs were introduced into the conventional IVIVE method. The CC was the inverse of the corresponding AFE. For control cases, the CC was 4.334 for CYP1A2, 5.369 for CYP2A6, 139.371 for CYP2B6, 18.938 for CYP2C8, 2.558 for CYP2C9, 24.026 for CYP2C19, 35.791 for CYP2D6, 4.152 for CYP2E1, and 0.540 for CYP3A4/5. For HCC patients, the CC was 11.524 for CYP2C19 and 0.474 for CYP3A4/5. Figure 2 shows that the corrected predictive hepatic clearance (CL’_H_) value for all CYPs for control cases had better accuracy than the CL_H_ values compared with observed clearance rates *in vivo*.

Based solely on clearance data for CYP2C19 and 3A4/5 in cirrhotic patients identified in a literature search, CCs that were suitable for controls and one that was calculated based on clearance in cirrhotic patients were introduced into the conventional method to analyze whether the CCs applied to controls were also suitable for HCC patients. Cross tabs with *χ*^2^ tests for independence analyses revealed that there were no significant differences between the HCC and control groups; results are shown in Table 1. From the examples of these two CYPs, one conclusion that might be tentatively drawn is that the CCs that were applied to control cases also were suitable for HCC patients.

**Table 1:** Comparison of the accuracy of predictions using different *in vivo* data

Drug	Group	Within a 2-fold error		Outside a 2-fold error		χ^2^ *P*-value
		Number	Percentage	Number	Percentage	
Omeprazole	Control	55	53.92	47	46.08	0.480
	HCC	60	58.82	42	41.18	
Midazolam	Control	95	93.14	7	6.86	1.000
	HCC	95	93.14	7	6.86	

Control: using a correction coefficient that applies to patients with normal liver function. HCC: using a correction coefficient calculated based on *in vivo* clearance in patients with cirrhosis.

The CL’_H_ values for CYPs in HCC patients are shown in Figure 1. The CL’_H_ values for CYP1A2, 2A6, 2B6, 2C8, 2C19, and 3A4/5 were significantly reduced (*P*=6.14E-13, 3.86E-6, 1.46E-3, 9.98E-27, 1.87E-9, and 1.30E-2, respectively). The most prominent reduction was observed for the CL’_H_ value for CYP2C8, which was reduced by 84.8%. Following CYP2C8 were CYP1A2, 2C19, 2B6, 2A6, 3A4/5, and 2C9, for which the CL’_H_ values were reduced by 60.7%, 57.2%, 43.9%, 38.0%, 19.6%, and 15.7%, respectively. Compared with controls, the CL’_H_ values for CYP2E1 and 2D6 increased by 40.3% and 11.9%, respectively, although it should be noted that the CL’_H_ values for CYP2C9 and 2D6 showed no significant differences.

For CL’_H_ values, obvious intra-individual variation could be observed. The maximum to minimum ratios were 83.9, 1036.7, 183.2, 147.4, 56.5, 73.8, 175.7, 60.6, and 11.5 for CYP1A2, 2A6, 2B6, 2C8, 2C9, 2C19, 2D6, 2E1 and 3A4/5, respectively; among these CYPs, CYP2A6 exhibited the greatest intra-individual variation.

### Bottom-up changes in CYP clearance

Figure 1 illustrates bottom-up changes in CYP clearance both *in vitro* and *in vivo* in patients with HCC compared with control patients. At each step, changes in the clearance values for CYPs differed. Notably, at each level (microsome, liver tissue, liver, and *in vivo*), CYP2E1 clearance was significantly increased, while the clearance values for CYP1A2, 2C8, and 2C19 were significantly reduced. The clearance values for CYP2A6, 2B6, and 3A4/5 showed no change at the microsomal level, although the clearance values at the other three levels were significantly reduced. The clearance values for CYP2C9 and 2D6 at the microsomal level were markedly increased, while those at the other three levels showed no change.

### Factors that impact the *in vivo* clearance

To further assess the effects of disease progression on *in vivo* clearance, HCC patients were categorized into fibrosis and cirrhosis subgroups based on their histological diagnosis. The results found that only the CL’_H_ for CYP2D6 was significantly different between the fibrosis and cirrhosis groups, and was increased by 88.3% in patients with cirrhosis (Figure 3A).

The impact of genetic polymorphisms on the *in vivo* clearance for CYPs was also assessed; no genetic influence and less than 7 polymorphic individuals were not displayed. As shown in (Figure 3B–3F), the CL’_H_ value for CYP1A2 was influenced by *CYP1A2* (*-163C>A*). The CL’_H_ values for CYP2B6 differed by the genotype of *CYP2B6 (516G>T)*. The CL’_H_ values for CYP2D6, 2E1, and 3A4/5 were influenced by *CYP2D6 (100C>T)*, *CYP2E1 (−333T>A)*, and *CYP3A5 (6986A>G)*, respectively.

The influence of gender, age, smoking and alcohol on the *in vivo* clearance for CYPs was also analyzed and found that smoking and alcohol consumption influenced the CL’_H_ for CYP2D6, as shown in (Figure 3G and 3H). Compared with nonsmokers, the CL’_H_ for CYP2D6 was significantly lower for smokers. Alcohol consumption significantly reduced the CL’_H_ for CYP2D6. Gender and age had no influence on the *in vivo* clearances for CYPs.

Together, our findings indicated that only CL’_H_ for CYP2D6 was easily influenced by disease progression, smoking and alcohol consumption, and only genetic mutations in *CYP1A2 (−163C>A)*, *CYP2B6* (*516G>T*), *CYP2D6* (*100C>T*), *CYP2E1* (*-333T>A*), and *CYP3A5* (*6986A>G*) had clear effects on the *in vivo* clearance for corresponding CYP.

## DISCUSSION

This represents the first extensive study to test the clearances for 10 CYPs *in vitro* and *in vivo* using 102 liver samples from patients with HCC accompanied by fibrosis or cirrhosis. We found that compared with controls the CL_M_ values increased for CYP2C9, 2D6 and 2E1, while the values for CYP1A2, 2C8 and 2C19 decreased and the values for CYP2A6, 2B6 and 3A4/5 were similar. MPPGL values corresponding with an unprecedented large number samples were first determined, which were significantly lower compared with controls [28.85 (7.60–93.60) mg/g *vs* 39.60 (9.90–127.90) mg/g] [10]. Based on changes in both physiological (e.g. MPPGL, LW, C_O_, and Q_H_) and enzyme kinetics (e.g. V_max_, K_m_ and CL_M_) parameters, the altered *in vivo* clearances for CYPs were identified. Compared with controls, the *in vivo* clearances for CYP1A2, 2A6, 2B6, 2C8, 2C19, 3A4/5 were significantly decreased, the clearance for CYP2E1 was markedly increased, while the clearances for CYP2C9 and 2D6 showed no change.

Using previously published data from other groups, an attempt to predict the pharmacokinetics of three drugs in liver cirrhosis had been performed [11]. Similarly, another previously attempt collected a series of parameters (e.g. CL_M_, V_max_, K_m_, and MPPGL) reported in the literature to predict the effects of liver cirrhosis on drug clearance [9]. Herein, we report the first attempt to predict the clearances for CYPs at different levels (e.g., liver tissue, liver, and *in vivo*) in HCC patients and to investigate the effects of HCC on the clearances for CYPs at different levels. Additionally, in our present study, we performed *in vitro* studies of each individual using 102 HLMs of HCC patients, determined the MPPGL of each individual, and recorded and saved other physiological and pathological parameters from each patient. Therefore, we could not only explore the reasons for these changes, but also revealed intra-individual variations in the clearances for CYPs at the microsome, liver tissue, liver, and *in vivo* levels.

Several previous studies have reported that the clearance values for several CYPs–CYP1A2, 2A6, 2D6, 2C19, 2E1 and 3A–were decreased [5, 12–17]. However, we found that the CL_M_ values for CYP2C9, 2D6 and 2E1 were significantly greater. This inconsistency could be due in part to the fact that these earlier studies focused on patients with simple cirrhosis who did not have accompanying HCC. Additionally, another study found that CYP2A6 clearance was reduced in patients with either moderate or severe alcoholic liver disease, but not in those with only mild disease [17]. By contrast, our present study showed that CYP2A6 clearances were not altered in the fibrosis or cirrhosis subgroups of HCC patients. These different characteristics identified in our present study suggest that a special reference standard may be essential for personalizing treatments of HCC patients with cirrhosis.

To the best of our knowledge, this present study was the first extensive to measure microsomal protein content in a large number of HCC patients. Compared with controls [39.60 (9.90–127.90) mg/g] [10], the MPPGL values [28.85 (7.60–93.60) mg/g] were significantly reduced in HCC patients (*P*=1.48E-5). Because of the reduced MPPGL, the CL_M_ for CYP2A6, 2B6, and 3A4/5 showed no change, but the CL_LT_ values were significantly reduced. The CL_M_ values for CYP2C9 and 2D6 were markedly increased, while that of CL_LT_ showed no change. Meanwhile, because of the markedly reduced LW in HCC patients, the CL_L_ was more significantly reduced compared with the CL_LT_. However, because of the elevated C_O_ and Q_H_ of HCC patients, the CL_H_ was less significantly reduced compared with the CL_L_. As a result of this bottom-up approach, a greater understanding of the changes in clearance values for CYPs at various levels in HCC patients became possible.

The effects of CYP genetic polymorphisms on the clearances for CYPs *in vivo* were analyzed and we found that only 5 of 24 mutations, including *CYP1A2 (−163C>A)*, *CTP2B6 (516G>T)*, *CYP2D6 (100C>T)*, *CYP2E1 (−333T>A)* and *CYP3A5 (6986A>G)* had clear effects on the clearances for CYPs *in vivo*.

To further verify the factors that influence CYPs clearances *in vivo* in HCC, we investigated the effects of general factors on CYP clearances. We found no obvious effect of gender and age on all CYPs clearances *in vivo*, no obvious effect of smoking and drinking on most CYP clearances, with the exceptions of CYP2D6. Although the CL’_H_ value for CYP2D6 were higher in non-smokers and non-drinkers, these factors were not likely to be related to clearance changes in patients. Indeed, the HCC group in this present study included more smokers and drinkers compared with the control group, and the CL’_H_ value for CYP2D6 for these patients were higher.

In summary, the bottom-up approach allowed us to predict pharmacokinetics in patients with HCC accompanied by fibrosis or cirrhosis and explored the reasons for changes in CYPs clearance at different levels. Because dosing recommendations are extremely limited and imprecise in HCC patients, this present study may provide important data to help clinicians to adjust their prescriptions for a wide range of drugs that are administrated to HCC patients. Because of the dramatically increased clearance for CYP2E1 in HCC patients, which metabolizes nitrosamine compounds into strong carcinogens, our observations may also aid further studies of the mechanisms of hepatocarcinogenesis in HCC patients. Because the clearance values *in vivo* for CYPs in the present paper is predicted, the accuracy of these predictions needs to be validated in future studies in HCC patients.

## MATERIALS AND METHODS

### Human liver samples

All non-tumor liver tissues were obtained from patients who had undergone surgical resection at Henan Provincal People's Hospital or Henan Provincal Tumor Hospital between March 2012 and July 2014; informed consent was obtained from each patient. Approvals for tissue collection and *in vitro* metabolism studies were obtained from the Medical Ethics Committee of Zhengzhou University. This research was conducted in accordance with the Declaration of Helsinki

HBV- or HCV-infected liver tissues (102 samples) were obtained from patients with HCC confirmed by postoperative pathological examination. Liver samples were classified as either having fibrosis or cirrhosis based on a histological diagnosis. As mentioned previously [18], 105 larger quantity of liver samples derived from 123 liver samples with normal function identified by histological diagnosis were collected from subjects with liver hemangioma, metastatic carcinoma, cholelithiasis, or gallbladder cancer for use as normal liver control tissues.

### Microsome preparation

Human liver microsomes (HLMs) were prepared by differential centrifugation as previously described [10]. Microsomal protein concentrations were measured using the Bradford method [19]. The MPPGL contents were determined as previously described [10].

### Clearance at microsomal level (CL_M_)

The drugs used for the enzymatic assays are probe substrates. Most CYP isoform-specific substrates and metabolite (7′-hydroxycoumarin, bupropion, hydroxybupropion, tolbutamide, 4′-hydroxytolbutamide, omeprazole, 5′-hydroxyomeprazole, dextromethorphan, O-demethylation dextrorphan, chlorzoxazone, 6-hydroxychlorzoxazone, and 1′-hydroxylation midazolam) were purchased from Sigma–Aldrich (St. Louis, MO, USA). Paclitaxel and 6-hydroxylpaclitaxel were purchased from Cayman Co. (Ann Arbor, MI, USA). Phenacetin, acetaminophen, midazolam and coumarin were purchased from the State Food and Drug Administration (Beijing, China). NADPH was obtained from Roche Co. (Basel, Switzerland). All organic solvents were of HPLC grade purity and were obtained from Siyou Chemical Reagent Co. (Tianjin, China).

Incubation mixtures contained HLMs (0.3 mg protein/ml for CYP1A2, 2A6, and 2E1; 0.2 mg protein/ml for CYP2D6 and 3A4/5; 0.5 mg protein/ml for 2B6, 2C8, 2C9, and 2C19), 100 mM phosphate buffer (pH 7.4) with 1mM NADPH and seven or eight concentrations of substrate (6.25-800μM for phenacetin, 0.156-20μM for coumarin, 7.8-500μM for bupropion, 2.5-80μM for paclitaxel, 31.25-2000μM for tolbutamide, 3.9-500μM for omeprazole, 0.625-960μM for dextromethorphan, 7.8-1000μM for chlorzoxazone, and 3.9-200μM for midazolam). The mixtures were pre-incubated for 5 min at 37°C. Optimal incubation times were as follows: 30 min for phenacetin O-deethylation, coumarin 7-hydroxylation, and chlorzoxazone 6-hydroxylation; 60 min for bupropion 4-hydroxylation, and tolbutamide 4-hydroxylation; 90 min for omeprazole 5-hydroxylation; 120 min for paclitaxel 6-hydroxylation; 20 min for dextromethorphan O-demethylation; and 5 min for midazolam 1′-hydroxylation. Reactions were terminated by adding 20 μl ice-cold acetonitrile or 1 ml ethylacetate or perchloric acid. Metabolites were identified by HPLC-UV or HPLC-FLD. The Michaelis–Menten constant (K_m_) and maximum reaction rate (V_max_) of each CYP were determined by nonlinear regression analysis using GraphPad Prism 6.0 (GraphPad Inc., La Jolla, CA, USA). CL_M_ was calculated based on the ratio of V_max_-to-K_m_.

### Genotyping

Genomic DNA was isolated from human liver tissues using a genomic DNA purification kit (QIAGEN Translational Medicine Co., Suzhou, China). Polymorphisms in 10 CYPs with frequencies of more than 1% in the Chinese sample set were investigated. A total of 20 allelic mutations determined by mass spectrometry and the alleles *CYP3A4* (*20230G>A*), *CYP1A2* (*-3860G>A*), and *CYP2B6* (*516G>T*) genotyped by PCR sequencing were performed by the LIUHE HUADA Genomics Technology Co., (Beijing, China). The alleles CYP2E1*1C/*1D were genotyped using two-step PCR.

### *In vivo* data collection

A PUBMED search or articles published from 1975 to 2015 was performed to collect information on the pharmacokinetics of 9 probe drugs for CYPs in humans; detailed information was summarized in Table 2. For the clearance of bupropion and chlorzoxazone, no “intravenous infusion” data was identified, so apparent oral clearance was used.

**Table 2:** Detailed data about 9 probe drugs for CYPs

CYP	Drug	Chemical Class	f_u, p_	R_B_	CL_in vivo_ (ml/min)
1A2	Phenacetin	N	0.594 [24]	1 [24]	1453.33±389.24 [25]
2A6	Coumarin	–	0.055	1	1602.5±547.9 [26]
2B6	Bupropion^a^	B	0.150 [27]	1 [27]	1112.25±280.26 [28]
2C8	Paclitaxel	–	0.098 [29]	0.69 [30]	496.42±210.48 [31]
2C9	Tolbutamide	A	0.056 [32–36]	0.55 [37]	19.36±10.60 [32–34]
2C19	Omeprazole	N	0.065 [34]	0.60 [38]	307.22±51.52 [39, 40]
2C19	Omeprazole^b^	N	0.065 [34]	0.60 [38]	67.0±40.0 [8]
2D6	Dextromethorphan	B	0.500 [34, 37]	0.55 [34, 37]	6471.67±5596.67 [41]
2E1	Chlorzoxazone^a^	B	0.028 [34]	0.55 [34]	131.42±40.08 [42–46]
3A4/5	Midazolam	N	0.042 [34, 47]	0.54 [34, 47]	426.65±95.37 [46–49]
3A4/5	Midazolam^b^	N	0.042 [34, 47]	0.54 [34, 47]	319.49±154.42 [7, 15]

N, neutral compound; B: basic compound; A: acidic compound; f_u, p_: fraction unbound in plasma; R_B_: ratio of the drug concentration in blood to plasma.

a because intravenous infusion method was identified in the search, apparent oral clearance was used orally administered drugs.

b data of drugs in cirrhotic patients.

If multiple published studies about the plasma unbound fraction (f_u, p_), blood-to-plasma concentration ratio (R_B_), and *in vivo* clearance for each drug, the overall mean for these data was used in this present paper. To determine the overall variability of clearance *in vivo* from multiple studies that reported the standard deviation (SD) for each drug, the overall SD was calculated.

The weighted mean (WX) was calculated using equation 1:

$$\text{WX}=\frac{\sum_{j=1}^{n}n_{j}\times x_{j}}{\sum_{j=1}^{n}n_{j}}$$ (Eq. 1)

where n_j_ is the number of observations and x_j_ is the mean value from the j^th^ study.

An overall SD was calculated from equations 2 and 3:

$$\text{Overall SD}=\sqrt{\frac{\text{Overall sum of squares}}{N}}$$ (Eq. 2)

$$\text{Overall sum of squares}=\sum_{j=1}^{n}\left[\left\{{\left(SD_{j}\right)}^{2}+{\left(x_{j}\right)}^{2}\right\}\times n_{j}\right]-N\times {\left(\text{WX}\right)}^{2}$$ (Eq. 3)

where N is the total number of observations from all studies and SD_j_ is the SD from the j^th^ study.

### Bottom-up calculations of hepatic clearance

For patients with HCC and controls, the bottom-up process using *in vitro* clearance to extrapolate *in vivo* clearance included several equations. In particular, the clearance for CYP in liver tissue (CL_LT_) was calculated using equation 4:

$$CL_{LT}=CL_{M}\times \text{MPPGL}$$ (Eq. 4)

The clearance for CYPs in liver (CL_L_) was determined using equation 5:

$$CL_{L}=CL_{LT}\times \text{LW}|\text{BW}$$ (Eq. 5)

where LW is liver weight and BW is body weight. According to the actual body weight given for each patient, the LW was calculated by multiplying the liver volume (LV) by the liver density, where LV (ml) = 12.5 × BW (kg) + 536.4 [20] and the liver density was 1.001 g/ml [21]. For HCC patients, pathology classifications indicated that 54 patients were without cirrhosis and 48 had early stage cirrhosis, so the LV of HCC patients was corrected by multiplying by the reciprocal of the fraction of the control LV (coefficient =1.12) [9].

The clearance for CYPs *in vivo* (CL_H_) was next calculated using the well-stirred model:

$$CL_{H}=\frac{Q_{H}\times CL_{L}\times f_{u,p}/R_{B}}{Q_{H}+CL_{L}\times f_{u,p}/R_{B}}$$ (Eq. 6)

where Q_H_ is determined as 24.5% [22] of the cardiac output (C_O_). C_O_ values originated from data for normal Han Chinese males (n = 783) and females (n = 805); mean values from each group were selected according to the age and gender of donors used in this study [23]. A study by Johnson *et al.* concluded that the Q_H_ in patients with Child–Pugh scores A for liver cirrhosis had no marked reduction [9]. Thus, the calculation of Q_H_ for HCC patients in this paper was the same as for controls.

Because individual CL_M_, MPPGL, BW, LW, and Q_H_ values (both HCC and control group) were used to predict clearance values for CYPs *in vivo*, the overall accuracy of the prediction was assessed based on the average fold-error (AFE), while the individual accuracy was assessed based on the individual fold-error (IFE). A two-fold bias limit corresponds to 0.5–2.0 of AFE and IFE values, which were estimated as follows:

$$\text{AFE}=10^{\frac{1}{\text{N}}\sum \text{log }(\text{predicted mean observed overall mean})}$$ (Eq. 7)

$$\text{IFE}=10^{\frac{1}{\text{N}}\sum \text{log }(\text{predicted individual value observed overall mean})}$$ (Eq. 8)

where N is the number of separate reports in the literature concerning intravenous drug clearance, except for bupropion and chlorzoxazone. Because there was no message about individual data in the literature, the observed overall mean was used as a reference was used to calculate the IFE.

To predict the CL_H_ for CYPs more accurately, a correction coefficient (CC) was introduced into the conventional *in vitro*–*in vivo* extrapolation (IVIVE) method. The CC of one CYP was the inverse of the AFE of the corresponding CYP.

### Statistical analysis

Most data sets were not normally distributed, so nonparametric methods were generally used for statistical analyses. The Mann–Whitney U test was used for pairwise comparisons. A *P*-value < 0.05 was considered to be statistically significant (two-tailed). SPSS statistics 17.0 software was used for statistical analyses. All graphs were generated using the Adobe Photoshop CC 2014, PowerPoint 2016 and GraphPad Prism version 6.0 software package.
